# Comparison of visual assessments of anisocytosis in canine blood smears and analyzer-calculated red blood cell distribution width

**DOI:** 10.3389/fvets.2023.1258857

**Published:** 2023-09-21

**Authors:** Asger L. Jensen, Anne K. H. Krogh, Lise N. Nielsen

**Affiliations:** Department of Veterinary Clinical Sciences, Faculty of Health and Medical Sciences, University of Copenhagen, Copenhagen, Denmark

**Keywords:** dog, hematology, microscopy, observer variability, erythrocyte size variability

## Abstract

Red blood cell distribution width (RDW) and visual assessments of anisocytosis assess variability in erythrocyte size. Veterinary studies on the correlation between the two methods and on observer agreement are scarce. The objectives were to assess the correlation of the grading of anisocytosis by means of conventional microscopy of canine blood smears to RDW, and to assess intra- and inter-observer variation in assessing the degree of anisocytosis. The study included 100 canine blood samples on which blood smear examination and RDW measurement were performed. RDW was measured on the Advia 2120i analyzer. The degree of anisocytosis was based on a human grading scheme assessing the ratio between the size of the representative largest red blood cell and that of the representative smallest red blood cell (1+ if <2x, 2+ if 2–3x, 3+ if 3–4x, and 4+ if >4x). Three observers participated and assessed the blood smears by conventional microscopy twice, 3 weeks apart by each observer. The correlation was assessed for each observer on each occasion using Kendahl-tau-b analysis. Intra-observer agreement was assessed using quadratically weighted kappa. Inter-observer agreement was assessed using free-marginal multi-rater kappa. Anisocytosis graded on blood smears correlated significantly with RDW values as assessed by Kendahl-tau-b ranging between 0.37 and 0.51 (*p* < 0.0001). Intra-observer agreement ranged from weak to moderate with resulting kappa-coefficients being 0.58, 0.68, and 0.75, respectively. Inter-observer agreement was weak (Kappa-values 0.44). The weak to moderate observer agreement in the visual assessment of anisocytosis indicates that the more precise and more repeatable RDW measurement should be used for clinical decision-making.

## 1. Introduction

Variability in erythrocyte size, anisocytosis, is useful for the describing, classifying, or prognosing of pathophysiological phenomena in dogs (1–4), such as anemia (5–9), microcytosis (10, 11), quatrefoil red blood cells (12), cardiopulmonary diseases (13–18), pancreatitis (19), inflammatory bowel disease (20), or diabetes mellitus (21), and of physiological phenomena such as sex and aging (22, 23).

The degree of anisocytosis is typically expressed either as variation in erythrocyte diameters in stained blood films (24) and/or by increased red cell distribution width (RDW) in automated analyzer counts (25). Evaluating a blood smear by conventional microscopy requires experience (26) and is prone to subjective assessment, which may lead to variability within and between observers. Furthermore, different grading schemes exist, some with very simple categorizations such as “absent or present” (9, 22). Others have applied subjective grading on a scale from +1 to +4, with +1 being the smallest amount (27), or grading +1 to +4 based on the number of variable-sized erythrocytes in a monolayer at 1,000x microscopic field (28) or high-power field (HPF) (21). Anisocytosis has even been graded by the number of microcytes visible in the standard field of view at × 100 magnification (11).

Red blood cell distribution width (RDW) is generated by most modern automated hematological analyzers from the distribution curve of erythrocyte volume derived by impedance changes, originally developed by Coulter (29, 30), and as seen in Idexx Procyte Dx (31), Sysmex pocH-100iV Diff (32), and in Abbot Sapphire, by flow cytometry and 2-angle laser light scatter, as present in instruments from Siemens such ad Advia2120 (33). In the SysmexXE5000, the size distribution of the erythrocytes is measured using the Sheath Flow Direct Current method where the signal amplitude changes when a blood cell passes through an aperture of the detector (34). The International Council for Standardization in Hematology (ICSH) (35) recommended standardization of the statistical method for analysis of erythrocyte volume distribution and for the algorithms used for the calculation of RDW in 1990. Seemingly, different manufacturers use different statistical methods and algorithms. A method to harmonize RDW by recalculating values according to instrument-specific polynomial curves has been suggested (36). Further, in some analyzers, RDW is calculated from the distribution histogram at the 50% height level above the baseline (Abbott); others use 20% height level above baseline (Sysmex XE500 and Mindray) or determine RDW from the erythrocyte volume histogram in the window between 60 and 120 fL (Siemens), with RDW being calculated as the ratio expressed in percentage between the standard deviation (SD) of erythrocyte volumes and the mean cell volume (MCV) (36, 37). In some analyzers, such as the Idexx Procyte Dx, RDW is reported as RDW-CV and RDW-SD, the difference being that RDW can be reported either as percentage (RDW-CV) or in femtoliters representing a standard deviation from the mean (RDW-SD). Irrespective of the method, RDW results below the reference interval should not be considered clinically meaningful, whereas an increased RDW value reflects a greater difference in the size of erythrocytes, which can be due to the presence of smaller and/or larger erythrocytes (36, 38).

In medical hematology, a study in 1975 did not show any strong relationship between anisocytosis graded on blood smears and analyzer-detected erythrocyte size variability, as only nine out of 40 patients with increased erythrocyte volume variability were identified as slightly or moderately anisocytic on blood smears (39). However, Simel et al. (40) found that semiquantitative grading of anisocytosis correlated with RDW (Kendal tau-b 0.22-0.40) and, when using intraclass correlation coefficient analysis, inter- and intra-observer agreement ranged between 0.336–0.553, and 0.400–0.642, respectively. Kumar et al. (41) also found high inter-observer agreement for semiquantitative grading of anisocytosis on blood smears (concordance correlation coefficient 0.73–0.76) but non-significant correlation between semi-quantitative grading of anisocytosis and RDW (concordance correlation coefficient 0.011). Finally, Jen et al. (42) found no intra-observer agreement (kappa coefficient 0.19) and no inter-observer agreement (kappa coefficient ranging between 0.16–0.19) in anemic patients.

In veterinary medicine, studies aiming to assess observer agreement and correlation between RDW and visual assessments of anisocytosis are rare. One study by Kumiega et al. (16) found that RDW results were within the reference range for dogs with degenerative mitral valvular disease while manual microscopic analysis of the blood smears revealed the presence of anisocytosis, although no direct correlation was reported. de Souza et al. (9) communicated that, in cases where anisocytosis was observed in blood smears in anemic dogs, these dogs also had higher RDW values and concluded that RDW values were more accurate than microscopic observations to detect anisocytosis.

As few veterinary studies have compared visual assessments of anisocytosis and RDW, the first research aim of this study was to evaluate whether anisocytosis assessed by conventional microscopy correlated to RDW. The second research aim was to evaluate observer variability in assessing the degree of anisocytosis using conventional microscopy. The research objectives were to assess the correlation of the semi-quantitative grading of anisocytosis by means of conventional microscopy of canine blood smears to RDW, and to assess intra- and inter-observer variation in assessing the degree of anisocytosis using conventional microscopy. The underlying rationale was that if semi-quantitative grading of anisocytosis correlated with RDW, the more precise of the two measures should be used for clinical decision-making.

## 2. Materials and methods

### 2.1. Study

The study was an observational, cross-sectional study on methodology, as the aim concerned method characteristics.

The Ethical Committee at the Department of Veterinary Clinical Sciences, SUND, University of Copenhagen approved the project (Approval number 2023-07), and all samples were anonymized.

### 2.2. Blood samples

In this study, only blood samples from dogs were included. This was due to the physical shape of canine RBC with biconcavity and there being a more pronounced central pallor in dogs compared to other common domestic animals (24). Dogs also have a larger array of erythrocyte morphologies compared to other species (43) and there was an availability of suitable blood samples at the laboratory.

All samples originated from privately owned dogs presented for diagnostic and/or therapeutic procedures at the University Hospital for Companion Animals, University of Copenhagen, where a hematologic analysis was requested by the attending veterinarian. This may result in the inclusion of samples with hemolysis and samples from both fasting and non-fasting dogs. However, hemolysis affect many hematologic parameters but not RDW when measured on Advia2120i (44). Further, in humans a light meal resulted in a small decrease 1 hour after eating (45). However, as the aim was to assess method characteristics and not relation to other patient characteristics, this does not affect the results of the present study.

Blood was collected in K_3_-EDTA-coated 2 mL blood collection tubes (Beckton-Dickinson Vacutainer). To avoid artifactual morphology changes as much as possible (46–48), all blood smears were prepared manually within 2 hours after arrival at the laboratory, using the wedge technique (49) and stained with modified Wright's stain in an automated stainer (Hematek 3000, Siemens Healthineers) or manually using Hemacolor (Merck, Denmark).

Blood samples were included irrespective of the final diagnoses, since the present study was a methodological study.

Red blood cell distribution width (RDW) on each blood sample was measured using an automated hematology system (ADVIA 2120i, Siemens Healthcare Diagnostics, Tarrytown, NY, USA), following the protocol and the canine setting in multispecies software provided by manufacturer. The analyzer was subjected to daily internal quality control and quarterly external quality control. In a prior internal evaluation of the analyzer, it was found that imprecision expressed by within- and between-run coefficients of variation (CV) ranged between 0.5–1.1% and 0.5–2.1%, respectively, based on replicate analysis of 10 canine blood samples with RDW within and above the established reference interval employed at the laboratory (11.7–14.3%). Over a 3-month period, approximately 15% of all canine samples analyzed had RDW values above the upper reference limit.

Koo and Li (50) suggest as a rule of thumb to obtain at least 30 heterogeneous samples. To secure a range of RDW values within and above the reference interval employed at the laboratory and a proportion of increased RDW values above the upper reference limit resembling daily practice (i.e., 15%), stored blood smears where RDW had been measured in the corresponding blood samples as well as prospectively collected blood samples were collected so that at least 15 blood smears with an increased RDW value were included. A total of 100 blood samples were thus included in the study.

Each blood smear was given an ID-number, and all slides were assessed on two separate occasions by each observer individually, 3 weeks apart. The order and ID numbers of the slides were changed randomly between the two occasions.

### 2.3. Observers

Koo and Li (50) further suggest to involve at least three observers when conducting reliability studies. Hence, the study included three observers with experience ranging from 10 to more than 30 years' post-graduate clinical pathological experience who routinely participate in the daily analysis of blood smears. Assessment of the impact of level of experience was not part of the aim of the study and thus not included in the study. In line with the recommendation by McHugh (51), all observers assessed five stored canine blood smears with different degrees of anisocytosis *in plenum* prior to the study using a multi-headed microscope to ensure that all observers agreed on the visual appearance of anisocytosis and applied the same microscopy and classification procedures.

### 2.4. Microscopy procedure

Evaluations were performed using Nikon Eclipse E200 (Nikon Corporation, Minato, Tokyo, Japan), equipped with an ocular CFI 10x/20, and objectives (Nikon E Plan 4× /0.1 ∞/– WD 30; Nikon E Plan 20× /0.40 ∞/0.17 WD 3.9; Nikon E Plan 50× /0.90 Oil ∞/× WD 0.35, and Nikon E Plan 100× /1.25 ∞/0.17 WD 0.23).

Each observer first selected the monolayer area of the blood smear using 40× or 200× magnification, the monolayer being identified as the area where approximately half of the erythrocytes touch one another, thus leaving out the lateral edges and the feathered edge. In cases of severe anemia where a monolayer could not be identified, the selected area was defined as the area on the blood smear where erythrocytes were separated by a distance of one cell diameter (28). Assessment of the degree of anisocytosis was then performed at 500×, as this allowed for a larger number of cells to be assessed in one view field. In case the individual observer needed to validate the observer's own assessment at 500×, this was performed at 1,000×.

### 2.5. Anisocytosis grading

The degree of anisocytosis was based on the grading scheme for human blood samples suggested by Gulati (52), where the ratio of the size of the representative largest red blood cell is compared to that of the representative smallest red blood cell, or assessed based on how many of the representative small red blood cells can fit into the representative largest red blood cell (1+ if <2×, 2+ if 2–3×, 3+ if 3-4×, and 4+ if >4× ). The method described by Gulati (52) was used to avoid introducing additional errors by defining and counting microcytes, normocytes, and macrocytes, and also to identify the representative largest and smallest erythrocytes over more than one view field. Typically, four to six fields were assessed to obtain an impression of the representative largest and smallest red blood cell.

In case an observer classified a blood smear to be of unacceptable quality, the blood smear was removed from the study and replaced by a new smear, which was evaluated as described.

### 2.6. Statistical analysis

The data for anisocytosis graded on blood smears were categorical on an ordinal scale and RDW values were on an interval scale.

For the assessment of the correlation between anisocytosis graded on blood smears and RDW values, correlations for each observer on each occasion between anisocytosis graded on blood smears and RDW values was assessed using Kendahl-tau-b analysis (53, 54). This procedure was selected as visual grading of anisocytosis in daily practice is done by one observer and not by a group of observers. The resulting Kendahl-tau-b correlation coefficients were interpreted as suggested by Khamis (55): 0.0-No relationship, 0.2-Weak positive relationship, 0.5-Moderate positive relationship, and 0.8-Strong positive relationship.

Intra-observer agreement was assessed for each observer using quadratically weighted kappa to take into account the disagreement between observations, such as a shift on the same blood smear from 1 to 2 being less serious than a shift from 1 to 3 (56, 57).

Inter-observer agreement was assessed using free-marginal multi-rater kappa as the study included three observers who were not restricted to assigning a certain number of cases to each category (58, 59).

To mitigate for the well-known effect of prevalence on Kappa-statistics (57), both stored blood smears where RDW had been measured in the corresponding blood samples as well as prospectively collected blood samples were included to obtain a proportion of increased RDW values above the upper reference limit resembling daily practice (15%).

Resulting kappa-values were interpreted as previously suggested (51): <0.20 no agreement, 0.21–0.39 minimal agreement, 0.40–0.59 weak agreement, 0.60–0.79 moderate agreement, 0.80–0.90 strong agreement, and >0.90 almost perfect agreement.

Statistical analyses were performed using the software MedCalc (60) with free-marginal kappa being calculated using an online kappa calculator (61).

## 3. Results

Of the 100 blood samples included in the study, none were classified as unacceptable by any of the observers. All observers noted that some (fewer than 15) blood smears were difficult to assess because of concomitant moderate to marked poikilocytosis or rouleaux formation. Because samples with poikilocytosis or rouleaux formation are likely to occur in daily practice, it was decided not to exclude these from the analysis to obtain a realistic as possible estimation of the observer variability in everyday practice. The underlying raw data set is available from the corresponding author.

**Figure 1:**
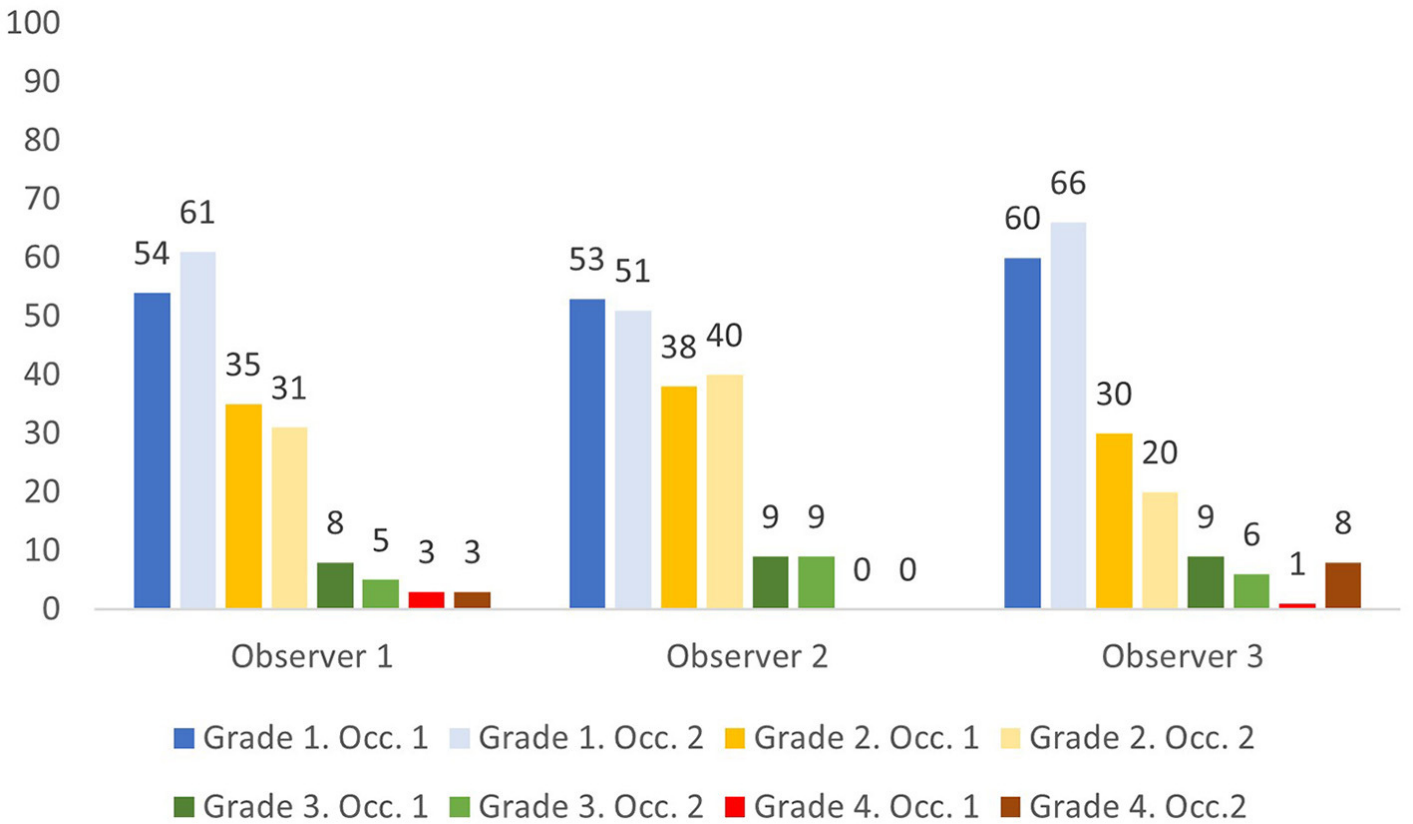
Percentage of samples assigned anisocytosis grade 1–4 by three observers on two occasions (Occ.1 and Occ. 2).

The 100 RDW values ranged from 10.6 to 22.1% with 16 values (16%) being above the upper reference limit for RDW of 14.3%. Overall, the three observers classified 51–61% as grade 1, 20–40% as grade 2, 5–9% as grade 3, and 0–8% as grade 4 (Figure 1). Of the 16 samples with RDW above 14.3%, the three observers classified between 0 and 2 cases as Grade 1, 4–7 cases as Grade 2, 4–8 cases as Grade 3, and 0–8 cases as Grade 4.

**Figure 2:**
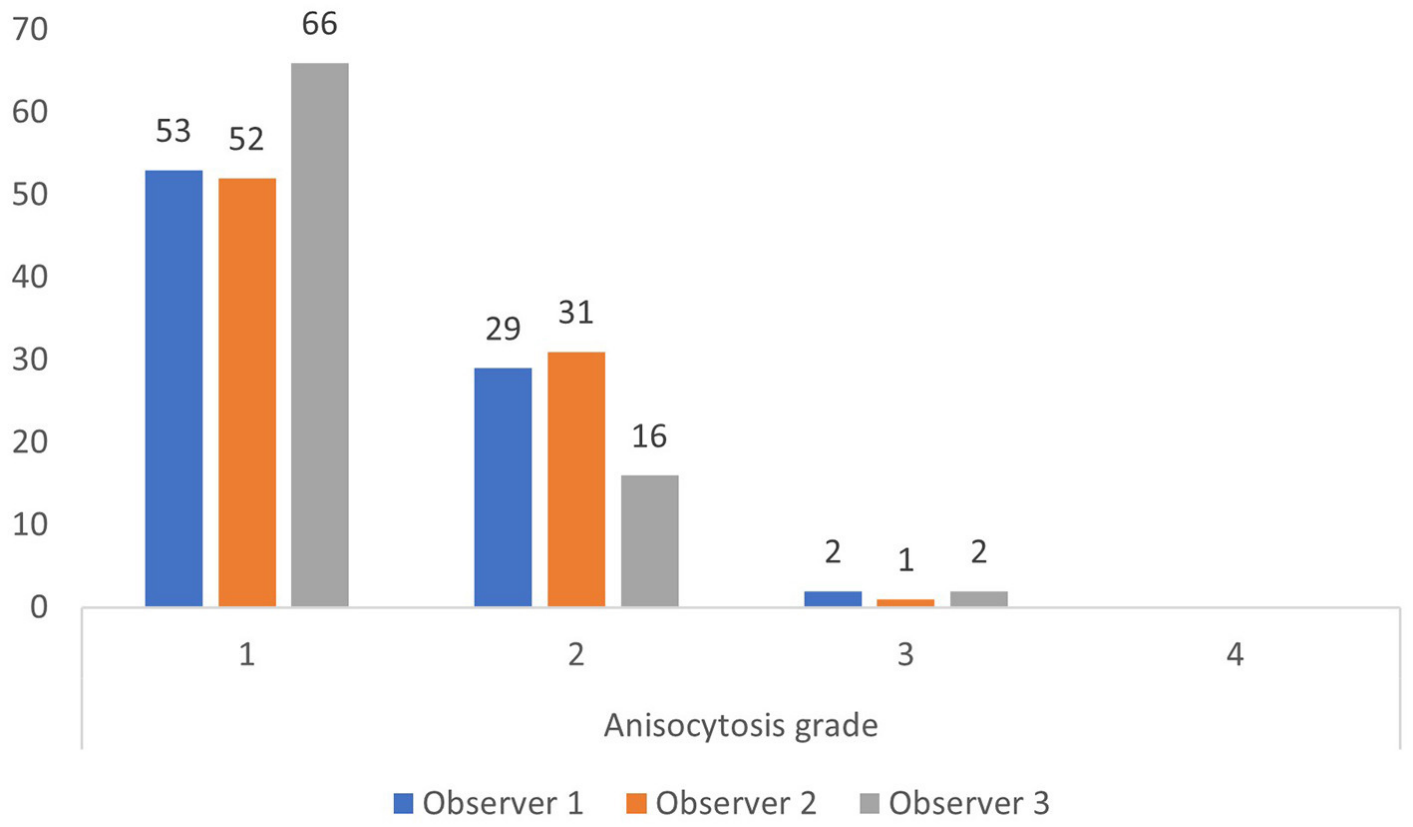
Anisocytosis grades by each observer when red cell density width (RDW) is below the upper limit of the reference interval of 14.3% (based on all 200 observations).

**Figure 3:**
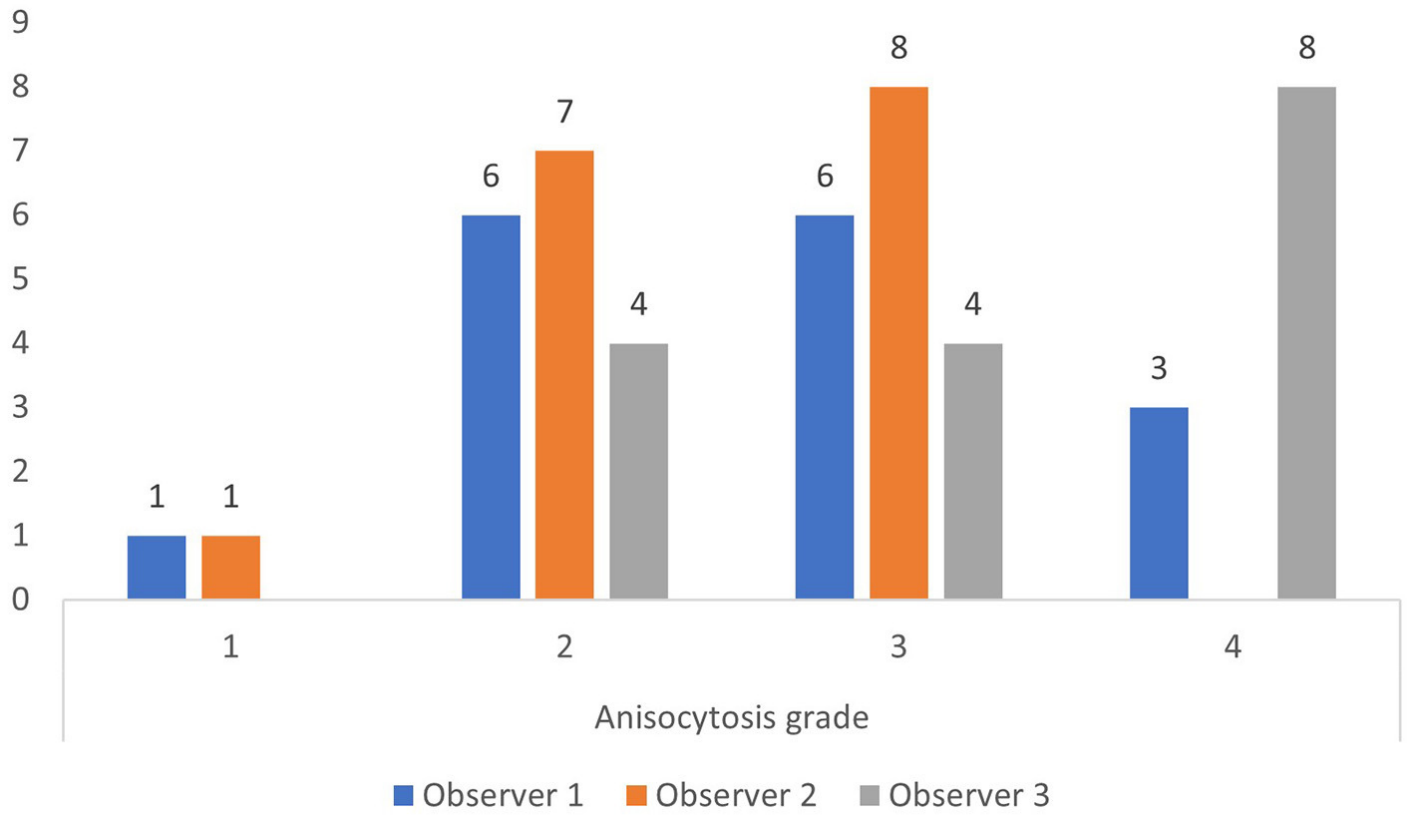
Anisocytosis grades by each observer when red cell density width (RDW) is above the upper limit of the reference interval of 14.3% (based on all 200 observations).

The upper reference limit for RDW is 14.3% at our laboratory. Using this limit, most observations by the three observers were anisocytosis grade 1 and 2 for RDW below 14.3%, and anisocytosis grade 2, 3, and 4 for RDW above 14.3% (Figures 2, 3).

**Figure 4:**
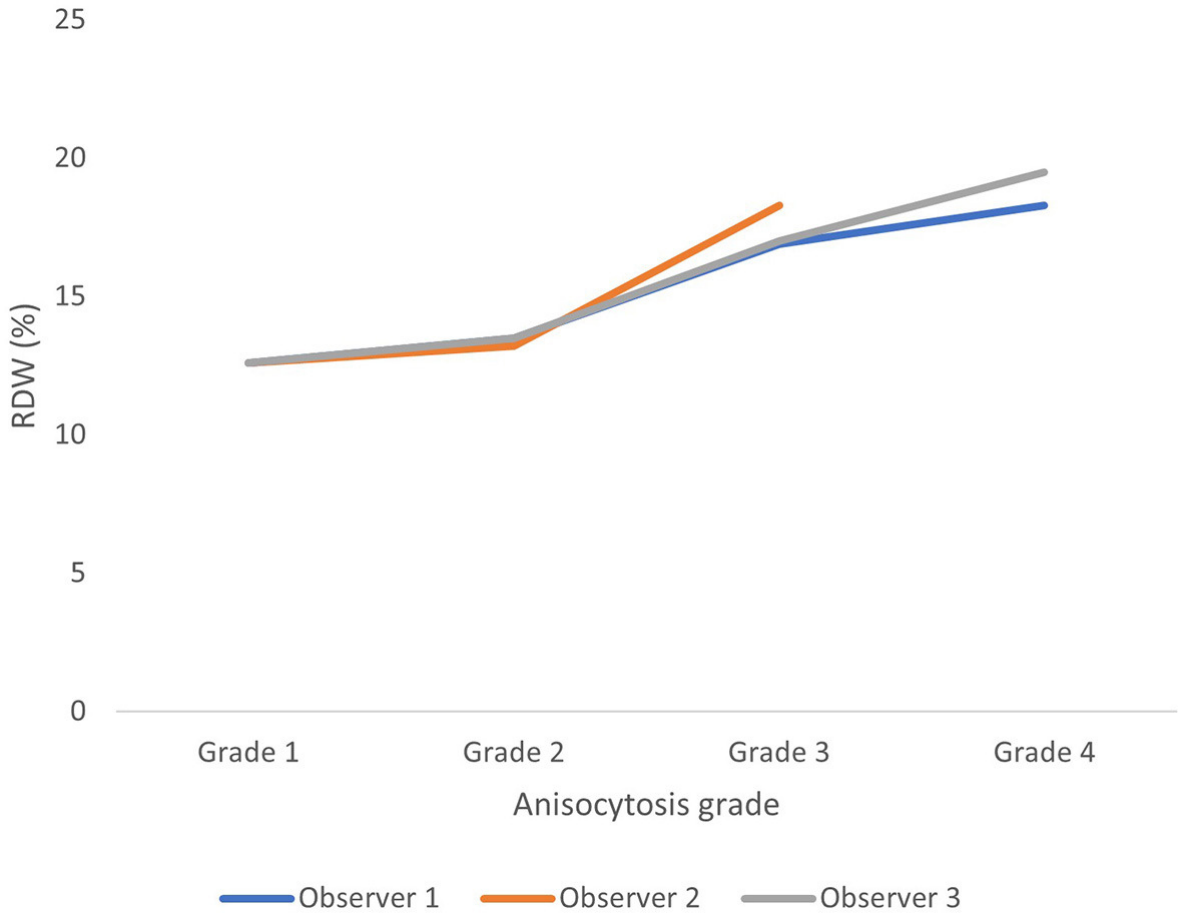
Average red blood cell density width (RDW) for each anisocytosis grade for each observer (based on all 200 observations). Anisocytosis graded on blood smears correlated moderately but significantly to RDW with Kendahl-tau-b ranging between 0.37 and 0.51 (*p* < 0.0001).

Anisocytosis graded on blood smears correlated moderately but significantly in this study to RDW with Kendahl-tau-b ranging between 0.37 and 0.51 (*p* < 0.0001) (Figure 4).

Intra-observer agreement as assessed by quadratically weighted kappa was weak to moderate, with resulting kappa-coefficients being 0.58, 0.68, and 0.75.

Inter-observer agreement as assessed by free-marginal kappa was weak. On the first occasion, the three observers agreed on classification on 39 occasions. Inter-observer variability expressed as kappa-value was 0.44 (0.36–0.53). On the second occasion, the three observers agreed on 39 occasions and inter-observer variability was 0.44 (0.35–0.53).

## 4. Discussion

RDW values are available in many veterinary laboratories, as is semiquantitative visual grading of anisocytosis. The two methods assess the same phenomenon, i.e., variability in erythrocyte size. In line with findings in medical hematology (40), the two methods also correlated in this study (Figure 2).

Visual microscopy to assess the degree of anisocytosis is associated with observer variability. In medical hematology, intra- and inter-observer agreement are moderate at best (39–42). This was also the case in the present study, where weak to moderate intra-observer agreement and weak inter-observer agreement were found. Several reasons for the weak to moderate observer agreement exist. For the semiquantitative assessment of anisocytosis, Gulati's method was applied in the present study to reduce additional errors from defining and assessing e.g., microcytes. However, in Gulati's method there are no solid borders between each category. For example, grade 2 includes RBC with a difference 2–3× and grade 3 includes a difference at 3–4×, thus if the degree of anisocytosis is borderline between the two grades, a variation is possible when assigning to one grade only.

Another reason for the weak to moderate observer variability is difficulties in assessing the degree of anisocytosis when poikilocytosis is present concomitantly. In this case, the Advia 2120i analyzer performs isovolumetric sphering of erythrocytes prior to analysis (33, 62, 63) and thus reduces poikilocytosis from RDW measurement, whereas the visual observer may experience difficulties in assessing the degree of anisocytosis. Also, marked rouleaux formation on blood smears may force visual assessments to be conducted nearer the feathered edge of the smear, potentially outside the monolayer. Although none of the observers excluded any samples as unacceptable, some samples that had either moderate to marked poikilocytosis or rouleaux formation were included to reflect everyday practice; this potentially could have interfered with observer variability, resulting in observer variability to be weak to moderate. Also, having each observer define the monolayer on each slide on each occasion made it very unlikely that the same field on each slide was evaluated by each observer on each occasion. This in turn adds to the magnitude of variability within and between observers. In contrast to the weak to moderate observer agreement found in this study, imprecision of RDW measurements on the Advia 2120i is below 2.5%. Seemingly, RDW measurement is more precise than visual assessment and thus, for clinical decision-making RDW values should be used. However, visual assessment of a blood smear remains a vital part of the CBC e.g., to validate the RDW value and other RBC features such as dimorphic RBC populations (64).

In the present study, only samples from dogs were included, and weak to moderate intra-observer agreement and weak inter-observer agreement were found. As intra- and inter-observer agreement in medical hematology are moderate at best (37–40), this could indicate that observer agreement is also weak to moderate in other animal species as well, thereby potentially also indicating that RDW values should be preferred over visual assessments of anisocytosis for clinical decision-making in other domestic animal species.

## Data availability statement

The raw data supporting the conclusions of this article will be made available by the authors, without undue reservation.

## Ethics statement

The animal studies were approved by the Ethical Committee at the Department of Veterinary Clinical Sciences, SUND, University of Copenhagen approved the project (Approval No. 2023-07), and all samples were anonymized. The studies were conducted in accordance with the local legislation and institutional requirements. Written informed consent was not obtained from the owners for the participation of their animals in this study because the study was approved by the Ethical Committee and no study on animals were conducted. Samples were already submitted and collected for diagnostic and/or therapeutic reasons so no additional samples were required. The samples were also anonymized and the study did not focus on the dogs included.

## Author contributions

AJ: Conceptualization, Investigation, Writing—original draft, Writing—review and editing. AK: Investigation, Writing—original draft, Writing—review, and editing. LN: Investigation, Writing—original draft, Writing—review, and editing.
